# Supplementary material for: Genome-Wide Association Study of Metamizole-Induced Agranulocytosis in European Populations
Source: Genes (Basel). 2020 Oct 29;11(11):1275. doi: 10.3390/genes11111275 (PMC7716224; doi:10.3390/genes11111275)
Supplement: Supplementary file 1 [file genes-11-01275-s001.zip › Supplementary Tables and Figures.pdf]

# *Supplementary Material*

## 1. Supplementary Tables

**Supplementary Table S1. Primers used for confirmation of rs111876221 genotypes in MIA/MIN-CH cohort.**

| Target region | Primer sequence (5’ - 3’) | Length (bp) | Annealing temperature (°Celsius) |
|---|---|---|---|
| *SERINC5* | F:TGGACAGCCCATAAGCCATC | 20 | 65.2 |
| | R1:GGTTGATATTCCTGTTTCTGATTGT | 25 | 65.2 |
| | R2: ATCCCAGTGTTCACCCAAACC | 21 | 65.2 |

**Supplementary Table S2: Pre-imputation data checking**. The pre-imputation checking program compared markers and their frequencies to the HRC reference panel (Human Reference Consortium r1-1 2016) to check for potential issues using both location information and SNP names. Variants with no match (name or position) to the reference or failing to meet one of the above thresholds were subsequently removed in PLINK. Mitochondrial (MT), X and Y-chromosomal (XY, Y) SNPs were excluded.

| Issue checked | Action performed |
| --- | --- |
| Chromosome and position | Updated |
| Non-matching alleles | Removed |
| XY, Y, MT SNPs | Removed |
| Allele frequency (AF) | Removed if AF difference > 0.2 |
| Strand | Updated |
| Palindromic SNPs | Removed if MAF > 0.4 |
| Reference allele | Updated |
| Variant naming | Updated |

**Supplementary Table S4. Characteristics of cases, tolerant and unexposed controls in the discovery cohort (MIA/MIN-CH).** Tolerant controls were patients treated with metamizole for at least 28 days without agranulocytosis or neutropenia.

| | **Cases N=45** | **Tolerant Controls N=38** | **Unexposed Controls N=153** |
|---|---|---|---|
| ANC < 500 /uL | 30 | - | - |
| Sex, male (%) | 23(51) | 7(45) | 72(47) |
| Age, years (%) | | | |
| <25 | 10(22) | 1(2.5) | 27(17.5) |
| 25-44 | 14(31) | 6(16) | 73(48) |
| 45-64 | 12(27) | 15(39.5) | 53(34.5) |
| 65-74 | 7(15.5) | 9(24) | - |
| >74 | 2(4.5) | 7(18) | - |
| BMI, median (range) | 24(19-47) | 28(16-39) | 23(18-40) |
| Latency time*[a] / treatment duration[b], days | 15(1-204) | 25 (5197) | NA |

[a] cases, [b] controls, * latency missing for 3 agranulocytosis cases and 1 neutropenia case.

ANC=lowest absolute neutrophil count, BMI=body mass index, NA=not applicable

**Supplementary Table 5. Top associations of the Phase I association analysis in the MIA/MIN-CH cohort.** Top genome-wide analysis results based on 195 genotyped SNPs in all 45 MIA/MIN cases vs. 191 controls (tolerant and unexposed). All results were adjusted for sex and four genetic multidimensional scaling (MDS) components. Chromosomal location is according to the Genome Reference Consortium human assembly GRCh37. CHR= chromosome, SNP=single nucleotide polymorphism, BP=base pair, MAF=minor allele frequency, OR [95%]=odds ratio with 95% confidence interval.

| CHR | SNP | Alleles (minor/major) | BP | MAF cases | MAF controls | OR [95%] | P-value | Gene region |
|---|---|---|---|---|---|---|---|---|
| 5 | rs10041917 | G/A | 79525305 | 0.24 | 0.42 | 0.44 [0.25-0.76] | $3.4x10^{-3}$ | *SERINC5* |
| 5 | rs7726099 | G/A | 79521704 | 0.25 | 0.43 | 0.46 [0.26-0.78] | $4.6x10^{-3}$ | *SERINC5* |
| 6 | rs4148876 | A/G | 32796793 | 0.17 | 0.65 | 2.62 [1.19-5.75] | $1.6x10^{-2}$ | *TAP2* |
| 12 | rs4149118 | G/A | 21011581 | 0.32 | 0.36 | 0.41 [0.19-0.86] | $1.8x10^{-2}$ | *SLCO1B3* |
| 5 | rs10036776 | A/G | 79509974 | 0.21 | 0.33 | 0.49 [0.27-0.89] | $1.9x10^{-2}$ | *SERINC5* |
| 5 | rs12522787 | A/G | 79526346 | 0.067 | 0.15 | 0.34 [0.13-0.90] | $3x10^{-2}$ | *SERINC5* |
| 6 | rs140017767 | G/T | 32813576 | 0.022 | 0.0026 | 16.3 [1.29-205.8] | $3x10^{-2}$ | *PSMB8/TAP2* |
| 6 | rs2071543 | T/G | 32811629 | 0.17 | 0.096 | 2.17 [1.05-4.47] | $3.5x10^{-2}$ | *PSMB8* |
| 6 | rs3823096 | T/C | 3014483 | 0.3 | 0.39 | 0.56 [0.32-0.97] | $3.9x10^{-2}$ | *NQO2* |
| 12 | rs10505871 | C/T | 21172053 | 0.25 | 0.38 | 0.50 [0.26-0.97] | $4.1x10^{-2}$ | *SLCO1B7* |

**Supplementary Table S6. Top associations of the Phase II association analysis in the MIA/MIN-CH cohort.** Top genome-wide analysis results based on 549 genotyped SNPs in all 45 MIA/MIN cases vs. 191 controls (tolerant and unexposed). All results were adjusted for sex and four genetic multidimensional scaling (MDS) components. Chromosomal location is according to the Genome Reference Consortium human assembly GRCh37. CHR= chromosome, SNP=single nucleotide polymorphism, BP=base pair, MAF=minor allele frequency, OR [95%]=odds ratio with 95% confidence interval.

| CHR | SNP | Alleles (minor/major) | BP | MAF cases | MAF controls | OR [95%] | P-value | Gene region |
|---|---|---|---|---|---|---|---|---|
| 5 | rs10041917 | G/A | 79525305 | 0.24 | 0.42 | 0.41 [0.24-0.71] | $1.7x10^{-3}$ | *SERINC5* |
| 5 | rs7726099 | G/A | 79521704 | 0.25 | 0.43 | 0.42 [0.24-0.73] | $2.2x10^{-3}$ | *SERINC5* |
| 5 | rs10036776 | A/G | 79509974 | 0.21 | 0.33 | 0.47 [0.25-0.85] | $1.3x10^{-2}$ | *SERINC5* |
| 5 | rs12522787 | A/G | 79526346 | 0.066 | 0.15 | 0.35 [0.14-0.86] | $2.3x10^{-2}$ | *SERINC5* |
| 5 | rs6895353 | G/A | 79514675 | 0.49 | 0.36 | 1.70 [1.07-2.71] | $2.3x10^{-2}$ | *SERINC5* |
| 12 | rs10505871 | C/T | 21172053 | 0.25 | 0.38 | 0.50 [0.27-0.92] | $2.7x10^{-2}$ | *SLCO1B7* |
| 6 | rs4148876 | A/G | 32796793 | 0.17 | 0.065 | 2.41 [1.10-5.26] | $2.7x10^{-2}$ | *TAP2* |
| 17 | rs11652446 | C/A | 72533076 | 0.42 | 0.34 | 1.84 [1.05-3.22] | $3.1x10^{-2}$ | *CD300C* |
| 6 | rs140017767 | G/T | 32813576 | 0.022 | 0.0026 | 15.17 [1.22-187.74] | $3.4x10^{-2}$ | *PSMB8/TAP1* |
| 6 | rs2071543 | T/G | 32811629 | 0.17 | 0.097 | 2.16 [1.04-4.49] | $3.8x10^{-2}$ | *PSMB8* |

**Supplementary Table S7. Top associations of the Phase I association analysis in the MIA-CH cohort.** Top genome-wide analysis results based on 195 genotyped SNPs in 30 MIA cases vs. 191 controls (tolerant and unexposed). All results were adjusted for sex and four genetic multidimensional scaling (MDS) components. Chromosomal location is according to the Genome Reference Consortium human assembly GRCh37. CHR= chromosome, SNP=single nucleotide polymorphism, BP=base pair, MAF=minor allele frequency, OR [95%]=odds ratio with 95% confidence interval.

| CHR | SNP | Alleles (minor/major) | BP | MAF cases | MAF controls | OR [95%] | P-value | Gene region |
|---|---|---|---|---|---|---|---|---|
| 5 | rs10041917 | G/A | 79525305 | 0.22 | 0.43 | 0.35 [0.18-0.70] | $2.7 \times 10^{-3}$ | *SERINC5* |
| 5 | rs7726099 | G/A | 79521704 | 0.23 | 0.43 | 0.38 [0.19-0.74] | $4.5 \times 10^{-3}$ | *SERINC5* |
| 6 | rs2228391 | C/T | 32797773 | 0.033 | 0.0026 | 377.7 [5.93-24028] | $5.1 \times 10^{-3}$ | *TAP2* |
| 6 | rs4148876 | A/G | 32796793 | 0.18 | 0.065 | 3.06 [1.37-6.83] | $6.1 \times 10^{-3}$ | *TAP2* |
| 5 | rs6895353 | G/A | 79514675 | 0.55 | 0.36 | 2.07 [1.18-3.62] | $1.1 \times 10^{-2}$ | *SERINC5* |
| 5 | rs10075871 | C/T | 79497415 | 0.63 | 0.45 | 2.05 [1.15-3.62] | $1.4 \times 10^{-2}$ | *SERINC5* |
| 6 | rs1044043 | A/C | 32793981 | 0.32 | 0.19 | 1.86 [1.03-3.35] | $3.8 \times 10^{-2}$ | *TAP2* |
| 5 | rs4704627 | T/C | 79488191 | 0.18 | 0.33 | 0.48 [0.23-0.96] | $3.9 \times 10^{-2}$ | *SERINC5* |
| 6 | rs17136117 | G/A | 3010337 | 0.033 | 0.0052 | 9.7 [1.09-86.35] | $4.2 \times 10^{-2}$ | *NQO2* |
| 5 | rs13355369 | T/C | 79518278 | 0.58 | 0.44 | 1.79 [1.01-3.15] | $4.4 \times 10^{-2}$ | *SERINC5* |

**Supplementary Table S8. Top associations of the Phase II association analysis in the MIA-CH cohort.** Top genome-wide analysis results based on 549 genotyped SNPs in 30 MIA cases vs. 191 controls (tolerant and unexposed). All results were adjusted for sex and four genetic multidimensional scaling (MDS) components. Chromosomal location is according to the Genome Reference Consortium human assembly GRCh37. CHR= chromosome, SNP=single nucleotide polymorphism, BP=base pair, MAF=minor allele frequency, OR [95%]=odds ratio with 95% confidence interval.

| CHR | SNP | Alleles (minor/major) | BP | MAF cases | MAF controls | OR [95%] | P-value | Gene region |
|---|---|---|---|---|---|---|---|---|
| 5 | rs10041917 | G/A | 79525305 | 0.22 | 0.43 | 0.35 [0.18-0.70] | $2.7x10^{-3}$ | *SERINC5* |
| 5 | rs7726099 | G/A | 79521704 | 0.23 | 0.43 | 0.38 [0.19-0.74] | $4.5x10^{-3}$ | *SERINC5* |
| 6 | rs2228391 | C/T | 32797773 | 0.033 | 0.0026 | 377.7 [5.93-24028] | $5.1x10^{-3}$ | *TAP2* |
| 6 | rs4148876 | A/G | 32796793 | 0.18 | 0.065 | 3.06 [1.37-6.83] | $6.1x10^{-3}$ | *TAP2* |
| 6 | rs6911119 | T/C | 160090193 | 0.05 | 0.0078 | 13.1 [1.99-86.4] | $7.3x10^{-3}$ | *SOD2* |
| 5 | rs6895353 | G/A | 79514675 | 0.55 | 0.36 | 2.07 [1.18-3.62] | $1.1x10^{-2}$ | *SERINC5* |
| 5 | rs10075871 | C/T | 79497415 | 0.63 | 0.45 | 2.05 [1.15-3.62] | $1.4x10^{-2}$ | *SERINC5* |
| 8 | rs10103029 | G/A | 18267338 | 0.033 | 0.17 | 0.17 [0.039-0.72] | $1.6x10^{-2}$ | *NAT2* |
| 12 | rs10841856 | C/T | 8692843 | 0.27 | 0.44 | 0.47 [ 0.25-0.87] | $1.7x10^{-2}$ | *CLEC4E* |
| 1 | rs6588432 | G/A | 53081321 | 0.3 | 0.17 | 2.25 [1.14-4.42] | $1.9x10^{-2}$ | *GPX7* |

**Supplementary Table S9. Top associations of the genome-wide association analysis in the MIA/MIN-CH cohort.** Top genome-wide analysis results based on 304,704 genotyped SNPs in all 45 MIA/MIN cases vs. 191 controls (tolerant and unexposed). All results were adjusted for sex and four genetic multidimensional scaling (MDS) components. Chromosomal location is according to the Genome Reference Consortium human assembly GRCh37. CHR= chromosome, SNP=single nucleotide polymorphism, BP=base pair, MAF=minor allele frequency, OR [95%]=odds ratio with 95% confidence interval

| **CHR** | **SNP** | **Alleles (minor/major)** | **BP** | **MAF cases** | **MAF controls** | **OR [95%]** | **P-value** | **Gene region** |
|---|---|---|---|---|---|---|---|---|
| 6 | rs191786 | C/T | 82497480 | 0.19 | 0.44 | 0.24 [0.13-0.45] | $8.6 \times 10^{-6}$ | - |
| 6 | rs9366076 | T/C | 167373708 | 0.4 | 0.17 | 3.57 [2.03-6.27] | $9.7 \times 10^{-6}$ | - |
| 9 | rs7865838 | G/A | 73109347 | 0.53 | 0.30 | 3.42 [1.97-5.94] | $1.1 \times 10^{-5}$ | *LOC101927086* |
| 7 | rs17152230 | C/T | 105506737 | 0.2 | 0.057 | 5.54 [2.54-12.03] | $1.5 \times 10^{-5}$ | *ATXN7L1* |
| 3 | rs79233583 | A/G | 86052689 | 0.12 | 0.021 | 10 [3.46-29.04] | $2.1 \times 10^{-5}$ | *CADM2* |
| 9 | rs957252 | C/A | 26059028 | 0.71 | 0.43 | 3.15 [1.85-3.4] | $2.4 \times 10^{-5}$ | - |
| 16 | rs6497573 | G/A | 22141042 | 0.47 | 0.24 | 2.88 [1.75-4.73] | $2.9 \times 10^{-5}$ | *VWA3A* |
| 4 | rs7667461 | A/G | 111622134 | 0.29 | 0.12 | 3.97 [2.08-7.61] | $3 \times 10^{-5}$ | - |
| 6 | rs162297 | G/A | 167455139 | 0.45 | 0.23 | 3.20 [1.84-5.55] | $3.4 \times 10^{-5}$ | *FGFR1OP* |
| 6 | rs194635 | C/T | 82521957 | 0.57 | 0.34 | 3.1 [1.81-5.28] | $3.5 \times 10^{-5}$ | - |

**Supplementary Table S10. Top associations of the genome-wide association analysis in the MIA-CH cohort.** Top genome-wide analysis results based on 304,704 genotyped SNPs in 30 MIA cases vs. 191 controls (tolerant and unexposed). All results were adjusted for sex and four genetic multidimensional scaling (MDS) components. Chromosomal location is according to the Genome Reference Consortium human assembly GRCh37. CHR= chromosome, SNP=single nucleotide polymorphism, BP=base pair, MAF=minor allele frequency, OR [95%]=odds ratio with 95% confidence interval.

| CHR | SNP | Alleles (minor/major) | BP | MAF cases | MAF controls | OR [95%] | P-value | Gene region |
|---|---|---|---|---|---|---|---|---|
| 6 | rs9366076 | T/C | 167373708 | 0.46 | 0.17 | 4.81 [2.49-9.30] | $2.9x10^{-6}$ | - |
| 9 | rs957252 | C/A | 26059028 | 0.78 | 0.43 | 4.73 [2.35-9.51] | $1.3x10^{-5}$ | - |
| 16 | rs9937453 | G/A | 22155629 | 0.4 | 0.15 | 3.87 [2.05-7.32] | $2.9x10^{-5}$ | *VWA3A* |
| 6 | rs162297 | G/A | 167455139 | 0.5 | 0.23 | 3.89 [2.05-7.37] | $3x10^{-5}$ | *FGFR1OP* |
| 4 | rs11098986 | A/G | 129700043 | 0.63 | 0.34 | 3.63 [1.97-6.65] | $3.1x10^{-5}$ | - |
| 1 | rs6687674 | A/G | 27537101 | 0.67 | 0.35 | 3.65 [1.98-6.73] | $3.3x10^{-5}$ | - |
| 17 | rs4968887 | T/C | 68264545 | 0.5 | 0.23 | 3.57 [1.93-6.57] | $4.3x10^{-5}$ | - |
| 9 | rs6475884 | A/C | 26121103 | 0.71 | 0.38 | 3.59 [1.93-6.66] | $5.2x10^{-5}$ | *LOC100506422* |
| 9 | rs7865838 | G/A | 73109347 | 0.56 | 0.30 | 3.98 [2.03-7.78] | $5.3x10^{-5}$ | *LOC101927086* |
| 4 | rs7667461 | A/G | 111622134 | 0.31 | 0.12 | 4.64 [2.18-9.86] | $6.3x10^{-5}$ | - |

**Supplementary Table S11. Replication of association signals from MIA/MIN-CH and MIA-CH analyses in the EuDAC cohorts.**
*imputed genotype; **imputed very poorly (RSQ=0.00024); NA, not available

| Cohort | CHR | Alleles (minor/major) | BP | MAF cases | MAF controls | OR [95%] | P-value | Gene region |
|---|---|---|---|---|---|---|---|---|
| EuDAC-ES | 5 | G/A | 79525305 | 0.53 | 0.41 | 1.0 [0.54-1.85] | 0.98 | *SERINC5* |
| EuDAC-DE | 5 | G/A | 79525305 | 0.41 | 0.50 | 0.70 [0.25-1.99] | 0.511 | *SERINC5* |
| EuDAC-ES | 6 | C/T | 82497480 | 0.38 | 0.41 | 0.86 [0.48-1.55] | 0.63 | *TENT5A* |
| EuDAC-DE | 6 | C/T | 82497480 | 0.29 | 0.30 | 0.94 [0.33-2.72] | 0.92 | *TENT5A* |
| EuDAC-ES | 1 | G/A | 53081321 | 0.17* | 0.12* | 1.52 [0.71-3.26] | 0.28 | *GPX7* |
| EuDAC-DE | 1 | G/A | 53081321 | 0.12 | 0.14 | 0.69 [0.16-3.01] | 0.63 | *GPX7* |
| EuDAC-ES | 6 | C/T | 32797773 | 0 | 0 | NA | NA | *TAP2* |
| EuDAC-DE | 6 | C/T | 32797773 | NA** | NA** | NA | NA | *TAP2* |
| EuDAC-ES | 6 | A/G | 32796793 | 0.069 | 0.055 | 1.15[0.33-3.94] | 0.68 | *TAP2* |
| EuDAC-DE | 6 | A/G | 32796793 | 0.125* | 0.098* | 1.51 [0.38-5.92] | 0.55 | *TAP2* |
| EuDAC-ES | 6 | T/C | 167373708 | 0.29* | 0.24* | 1.36 [0.69-2.66 | 0.36 | *RNASET2* |
| EuDAC-DE | 6 | T/C | 167373708 | 0.25 | 0.23 | 1.39 [0.45-4.36] | 0.56 | *RNASET2* |

## 2. Supplementary Figures

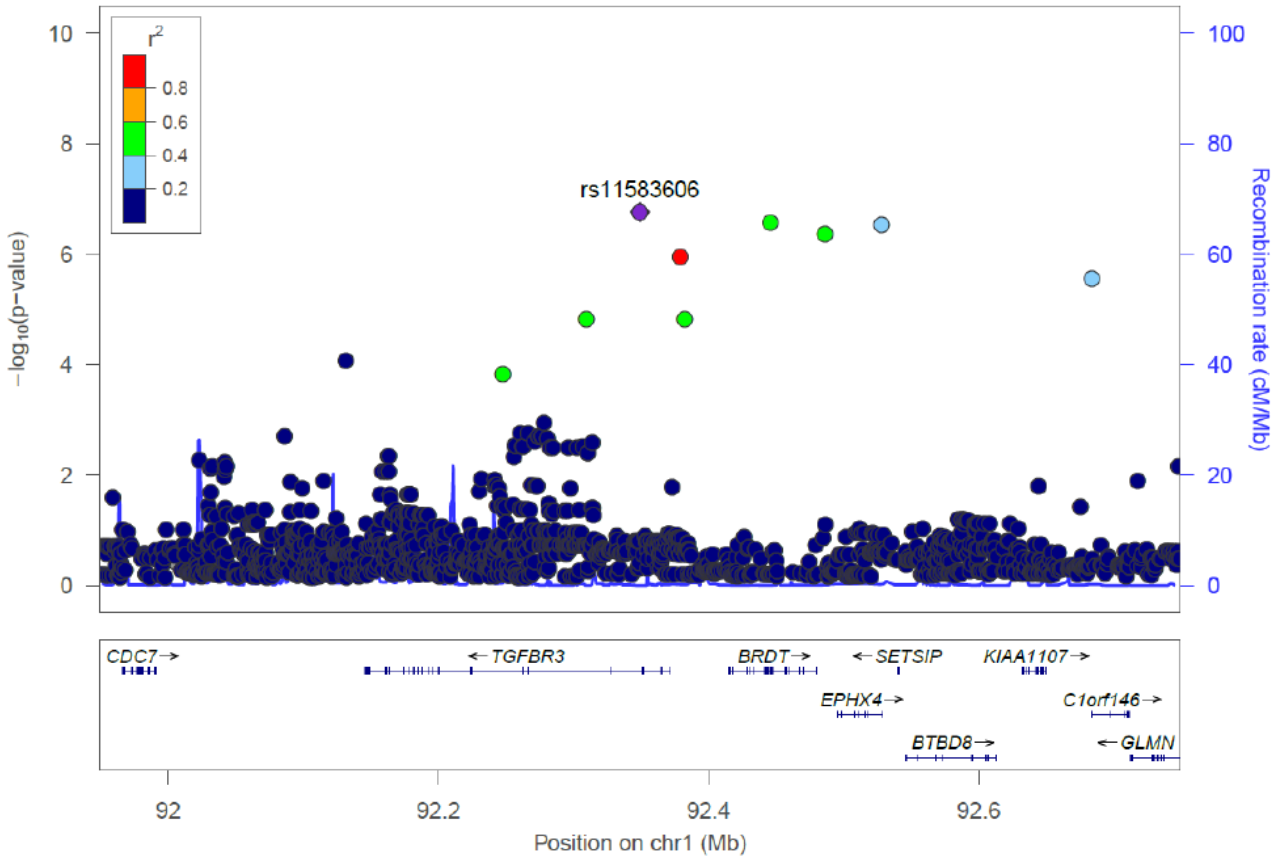


**Supplementary Figure S1. Regional association plot for regions around rs11583606.** Plot was produced in Locus Zoom and shows the most strongly associated SNP from the GWAS meta-analysis of all 86 cases versus 464 controls. Different colors represent the strength of the LD of each SNP with the most significant SNP represented by a diamond.

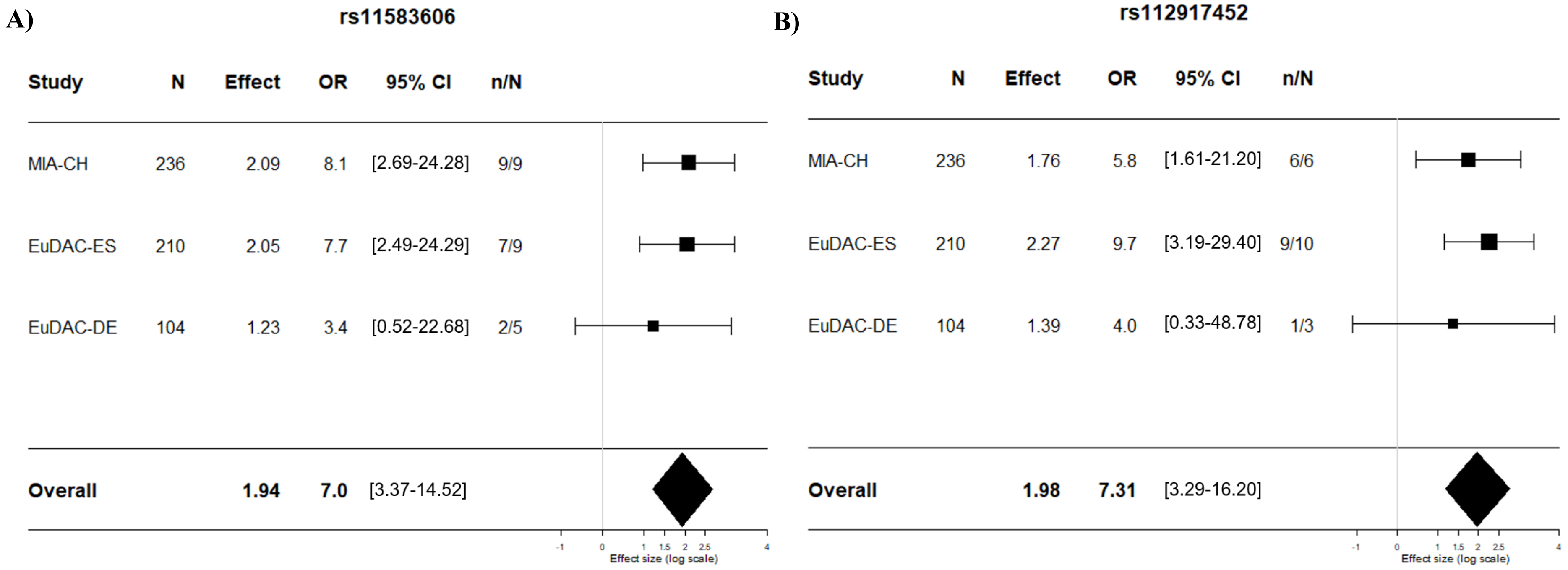


**Supplementary Figure S2. Forest plot of associations with metamizole-induced agranulocytosis and neutropenia in the three independent cohorts (all cases and controls).** Estimated effect sizes, odds ratio (OR) with 95% confidence intervals (CI), P-value for the independently associated rs11583606 (A) and rs112917452 (B) in 86 MIN and MIA cases and 464 controls (tolerant and unexposed). The effects are adjusted for sex and genetic multidimensional scaling (MDS) components 1-4. Numbers of patients (n) or controls (N) are given in the n/N column. N=sample size.

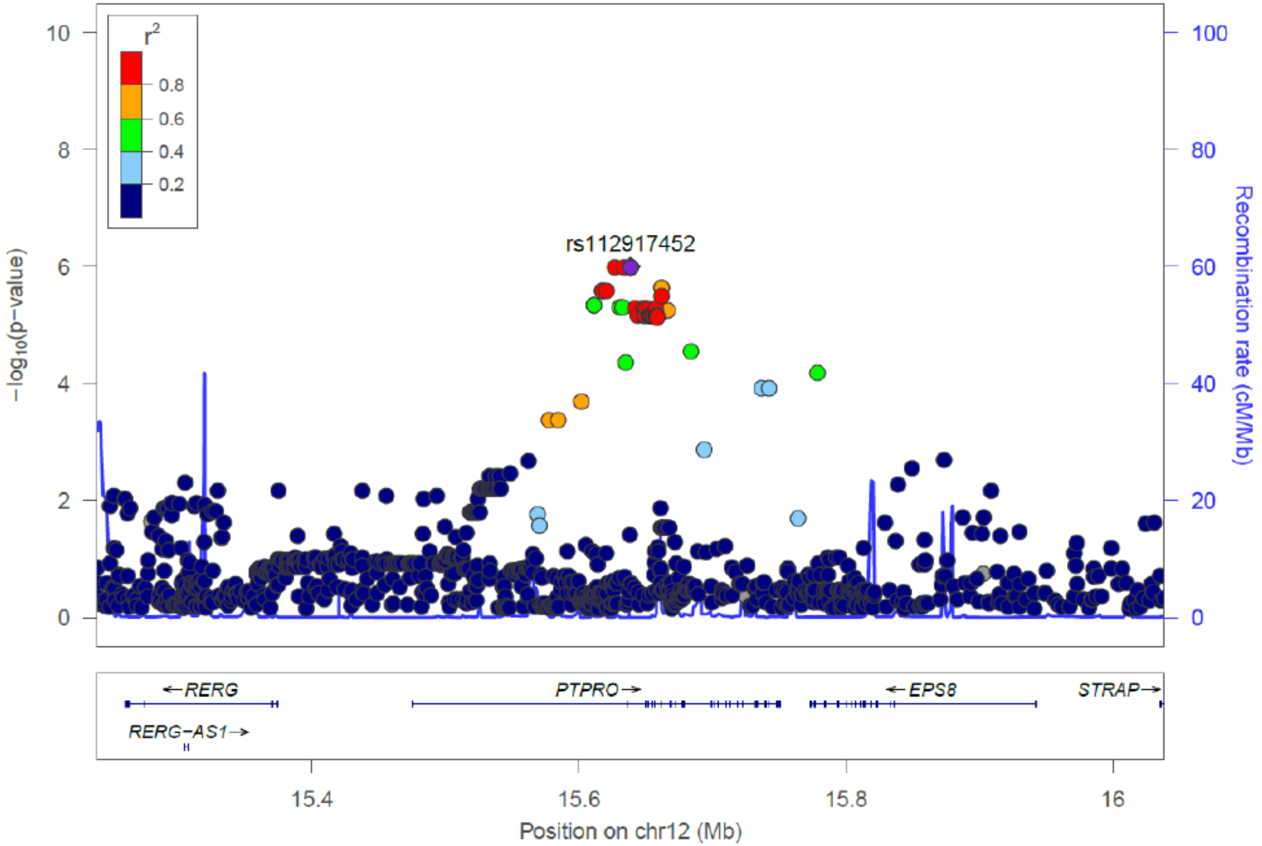


**Supplementary Figure S3. Regional association plot for regions around rs112917452.** Plot was produced in Locus Zoom and shows the most strongly associated SNP from the GWAS meta-analysis of all 86 cases versus 464 controls. Different colors represent the strength of the LD of each SNP with the most significant SNP represented by a diamond.

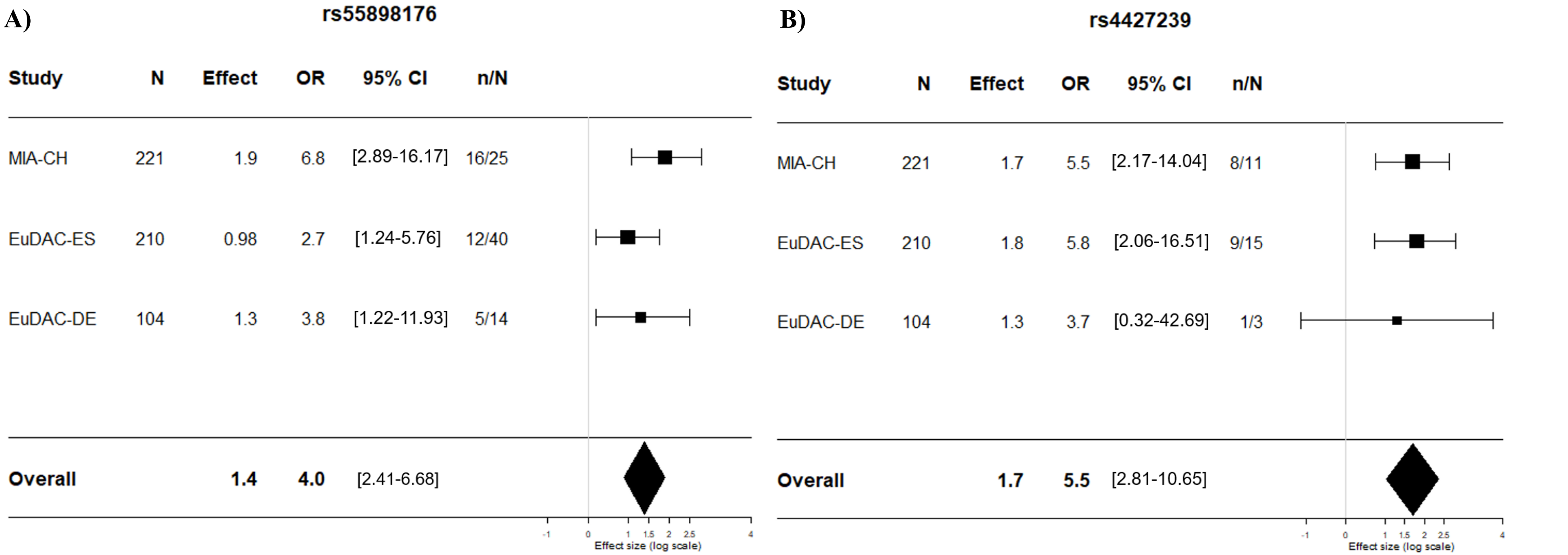


**Supplementary Figure S4. Forest plot of associations with metamizole-induced agranulocytosis in the three independent cohorts (agranulocytosis cases and controls).** Estimated effect sizes, odds ratio (OR) with 95% confidence intervals (CI), P-value for the independently associated rs55898176 (A) and rs4427239 (B) in 71 MIAcases and 464 controls (tolerant and unexposed). The effects are adjusted for sex and genetic multidimensional scaling (MDS) components 1-4. Numbers of patients (n) or controls (N) are given in the n/N column. N=sample size.

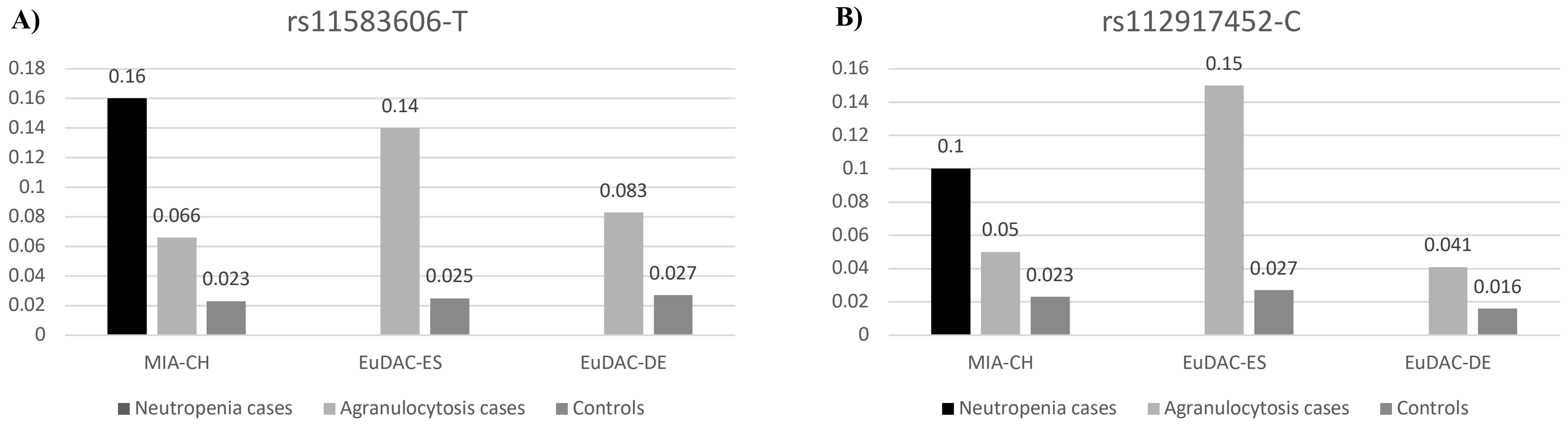


**Supplementary Figure S5. Allele frequency distribution of the leading SNPs associated with metamizole-induced agranulocytosis and neutropenia in the three independent cohorts (all cases versus controls).** The allele frequencies show the risk allele distribution of rs11583606 (A) and rs112917452 (B) in the MIN (black), MIA (light grey) and control groups (dark grey) of the MIA/MIN-CH, EuDAC-ES and EuDAC-DE cohorts.

A)

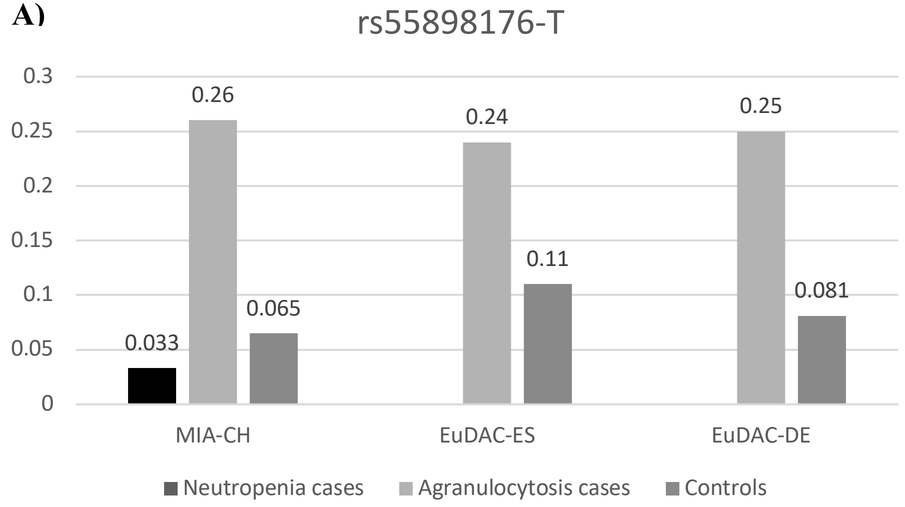


B)

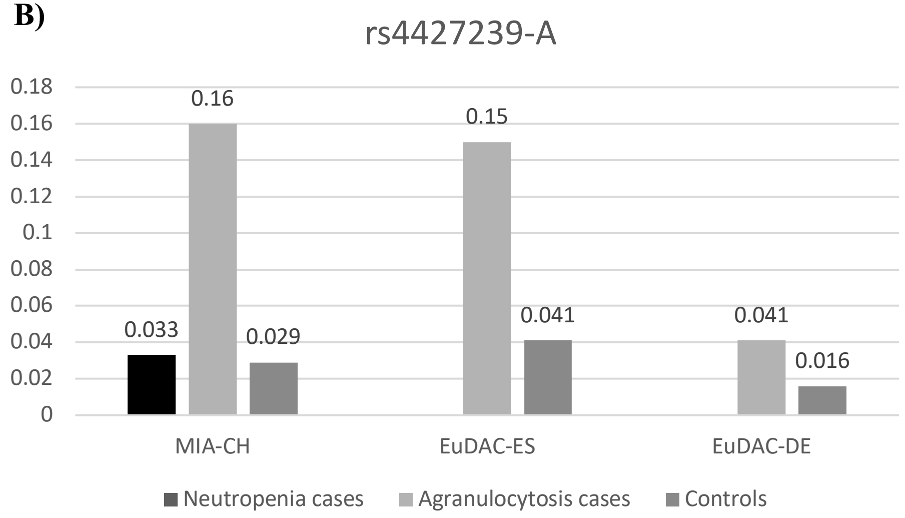


**Supplementary Figure S6. Allele frequency distribution of the leading SNPs associated with metamizole-induced agranulocytosis in the three independent cohorts (agranulocytosis cases versus controls).** The allele frequencies show the risk allele distribution of rs55898176 (A) and rs4427239 (B) in the MIN (black), MIA (light grey) and control groups (dark grey) of the MIA/MIN-CH, EuDAC-ES and EuDAC-DE cohorts.
